# *Torquenema* n. g., *Wallabicola* n. g., and *Macropostrongyloides phascolomys* n. sp.: New Genera and a New Species of Nematode (Strongylida: Phascolostrongylinae) Parasitic in Australian Macropodid and Vombatid Marsupials

**DOI:** 10.3390/ani11010175

**Published:** 2021-01-13

**Authors:** Tanapan Sukee, Ian Beveridge, Abdul Jabbar

**Affiliations:** Department of Veterinary Biosciences, Melbourne Veterinary School, Faculty of Veterinary and Agricultural Sciences, The University of Melbourne, Melbourne, VIC 3030, Australia; jabbara@unimelb.edu.au

**Keywords:** parasites, Phascolostrongylinae, kangaroos, wallabies, wombats, marsupials, taxonomy

## Abstract

**Simple Summary:**

This article describes two new genera of parasitic nematodes, *Torquenema* n. g. from the eastern grey kangaroo and *Wallabicola* n. g. from the swamp wallaby, and a new species, *Macropostrongyloides phascolomys* n. sp. from the caecum of the common wombat.

**Abstract:**

The strongyloid nematodes belonging to the subfamily Phascolostrongylinae occur primarily in the large intestines of macropodid and vombatid marsupials. Current molecular evidence suggests that the two nematode species, *Macropostrongyloides dissimilis* and *Paramacropostrongylus toraliformis*, from macropodid marsupials are distant from their respective congeners. Furthermore, specimens of *Macropostrongyloides lasiorhini* from the large intestines of the southern hairy-nosed wombat (*Lasiorhinus latifrons*) and the common wombat (*Vombatus ursinus*) are genetically distinct. This study aimed to describe the new genera *Torquenema* n. g. (with *T. toraliforme* n. comb. as the type species) from the eastern grey kangaroo, *Wallabicola* n. g. (with *W. dissimilis* n. comb. as the type species) from the swamp wallaby and a new species *Macropostrongyloides phascolomys* n. sp. from the common wombat, using light and scanning electron microscopy.

## 1. Introduction

The strongyloid nematode subfamily, Phascolostrongylinae Lichtenfels, 1980 is currently represented by seven genera and 19 species found in kangaroos, wallabies, and wombats (Macropodidae and Vombatidae) [1]. The Phascolostrongylinae has been subdivided into three tribes based on morphological features [1]. One of the tribes is the Macropostrongyloidinea, which comprises the genera *Macropostrongyloides* Yamaguti, 1961 and *Paramacropostrongylus* Johnston and Mawson, 1940, and they are generally characterised by small cylindrical buccal capsules with a distinctive Y-shaped dorsal gutter and the presence of peri-oral teeth or denticles [1]. Although the majority of species within the Phascolostrongylinae are restricted to the large intestines of their hosts, several exceptional species, all belonging to the tribe Macropostrongyloidinea, have been found within the stomachs of macropodid marsupials [2]. 

A recent molecular study based on the first and second internal transcribed spacers (ITS-1 and ITS-2) of the nuclear ribosomal DNA revealed that the genera *Macropostrongyloides* and *Paramacropostrongylus* were paraphyletic, with *Macropostrongyloides dissimilis* Johnston and Mawson, 1939 and *Paramacropostrongylus toraliformis* Beveridge and Mawson, 1978 distinct from their respective congeners [3]. The study found that *M. dissimilis*, a dark-red nematode from the stomach of the swamp wallaby *Wallabia bicolor* (Desmarest) is a sister taxon to the only other stomach-inhabiting phascolostrongyline species *Paramacropostrongylus typicus* Johnston and Mawson, 1940 and *Paramacropostrongylus iugalis* Chilton, Beveridge and Andrews, 1993 from grey kangaroos [3]. 

*Macropostrongyloides dissimilis* was originally assigned to the genus *Cyclostrongylus* Johnston and Mawson 1939, but a subsequent revision found that the species shared greater similarities with its current genus, *Macropostrongyloides* [4]. *Paramacropostrongylus toraliformis* was described from the caecum of the eastern grey kangaroo, *Macropus giganteus* Shaw. This species differed at that time from the only congener, *Paramacropostrongylus typicus* in the presence of a prominent cephalic collar which extends beyond its body [2]. 

*Macropostrongyloides lasiorhini* Mawson, 1955 is the only species of *Macropostrongyloides* parasitic in the large intestine of wombats. Molecular data also detected genetic differences between nematodes from the southern hairy-nosed wombat, *Lasiorhinus latifrons* (Owen) and the common wombat, *Vombatus ursinus* (Shaw) [5].

## 2. Materials and Methods 

### 2.1. Collection of Specimens

Specimens were borrowed from the Australian Helminthological Collection, South Australian Museum (SAM), Adelaide and the Australian National Wildlife Collection (ANWC), Commonwealth Scientific and Industrial Research Organisation (CSIRO), Canberra, or collected from road killed hosts and stored in ethanol at −80 °C until required. These latter specimens have now been deposited in SAM. 

### 2.2. Light Microscopy 

Specimens were mounted on glass slides in lactophenol and examined under a compound microscope (Olympus BH-2, Olympus Optical CO., LTD., Tokyo, Japan). Illustrations were made using a drawing tube attached to a BH-2 Olympus microscope and digitised in Adobe Photoshop CC 2019. Measurements are presented in millimetres as ranges followed by the mean in parenthesis. The lengths and widths of the buccal capsule were taken from the external surface. 

Morphological terminology follows that of Beveridge and Mawson [2] except for the papillae of the genital cone which follows the numerical system of Chabaud et al. [6]. Host nomenclature follows Jackson and Groves [7]. In instances where collection localities were within approximately 50 km of one another, records of occurrences were combined.

### 2.3. Scanning Electron Microscopy 

Specimens for scanning electron microscopic (SEM) examination were dehydrated in an ethanol series with concentrations beginning at 70%, followed by 1:1 ratio of ethanol and hexamethyldisilazane (HMDS) (ProSciTech Pty. Ltd., Queensland, Australia) and finally with pure HMDS. The dehydrated specimens were air-dried and mounted onto stubs with double-sided carbon conductive tape, coated in gold and observed in a FEI XL30 FEG scanning electron microscope (Thermo Fisher Scientific Inc., Massachusetts, MA, USA).

## 3. Results

### 3.1. Description of a New Genus, Torquenema n. g.

Order Strongylida (Molin, 1861)

Family Chabertiidae (Popova, 1952)

Subfamily Phascolostrongylinae Lichtenfels, 1980

*Torquenema* n. g.

*Type species*: *Torquenema toraliforme* (Beveridge and Mawson, 1978) n. comb.

#### 3.1.1. Generic Definition

Robust nematodes. Cephalic collar large, disc-shaped, extending peripherally beyond body; collar pierced by four sub-conical, sub-median papillae and two lateral amphids; mouth opening circular; buccal capsule sub-cylindrical, heavily sclerotised; anterior extremity bears c.25 triangular projections forming leaf crown; teeth absent in buccal capsule; duct of dorsal oesophageal gland pierces wall of buccal capsule at mid-length, divides internally to form Y-shaped dorsal gutter. Oesophagus clavate; nerve ring in anterior third of oesophagus; deirid papillate, posterior to nerve ring; excretory pore at same level as deirid. Spicules elongate, alate. Vulva immediately anterior to anus; ovejector Y-shaped; eggs ellipsoidal. Parasitic in macropodid marsupials.

#### 3.1.2. Redescription of *Torquenema toraliforme* n. comb.

*Torquenema toraliforme* (Beveridge and Mawson, 1978) n. comb.

*Synonyms: Paramacropostrongylus toraliformis* Beveridge and Mawson, 1978.

*Types*: holotype, SAM 41299; allotype, SAM 41300. 

*Type host*: *Macropus giganteus* Shaw (Marsupialia: Macropodidae).

*Type locality*: Yan Yean, Victoria, Australia (37°34′ S 145°06′ E).

*Additional material examined*: Queensland: 1♂, 30 km E of Inglewood (SAM 33088), 1♂, Warwick (SAM 49053); New South Wales: 2♂♂, Girilambone (SAM 33572); 10♂♂, 12♀♀, Kingstown (SAM 6730); 3♀♀, Armidale (SAM 6765); 4♂♂, 3♀♀, 45 km NE of Coonabarabran (SAM 44383); 4♂♂, 3♀♀, Kioloa (SAM 48486); Victoria: 32 ♂♂, 62♀♀, Dartmouth (SAM 9209, 9244); 1♀, Mitta Mitta (SAM 12060); 8♂♂, 1♀, Fraser National Park (SAM 11049); 1♀, Boho South (SAM 34627); 4♂♂, 4♀♀, Cardinia (SAM 22954); 3♂♂, 3♀♀, Yarra Glen (SAM 46172); 10♂♂, 20♀♀, Yan Yean (SAM 9614); 2♂♂, 3♀♀, Craigieburn (SAM 48496); 10♂♂, 12♀♀, Lara (SAM 33122); 2♂♂, 5♀♀, Anglesea (SAM 44435).

*Site in host*: Caecum.

*Representative DNA sequences:* Ribosomal DNA first and second internal transcribed spacers sequences (Genbank Accession MT396206) from *M. giganteus*, Research, Victoria, Australia [3]. 

*Etymology*: The new generic name is derived from ‘torquem’ which means collar in Latin and refers to the prominent cervical collar of *Torquenema* n. g. The specific name has been changed to *toraliforme* n. comb. in agreement with the neuter generic name.

#### 3.1.3. Redescription 

**Figure 1:**
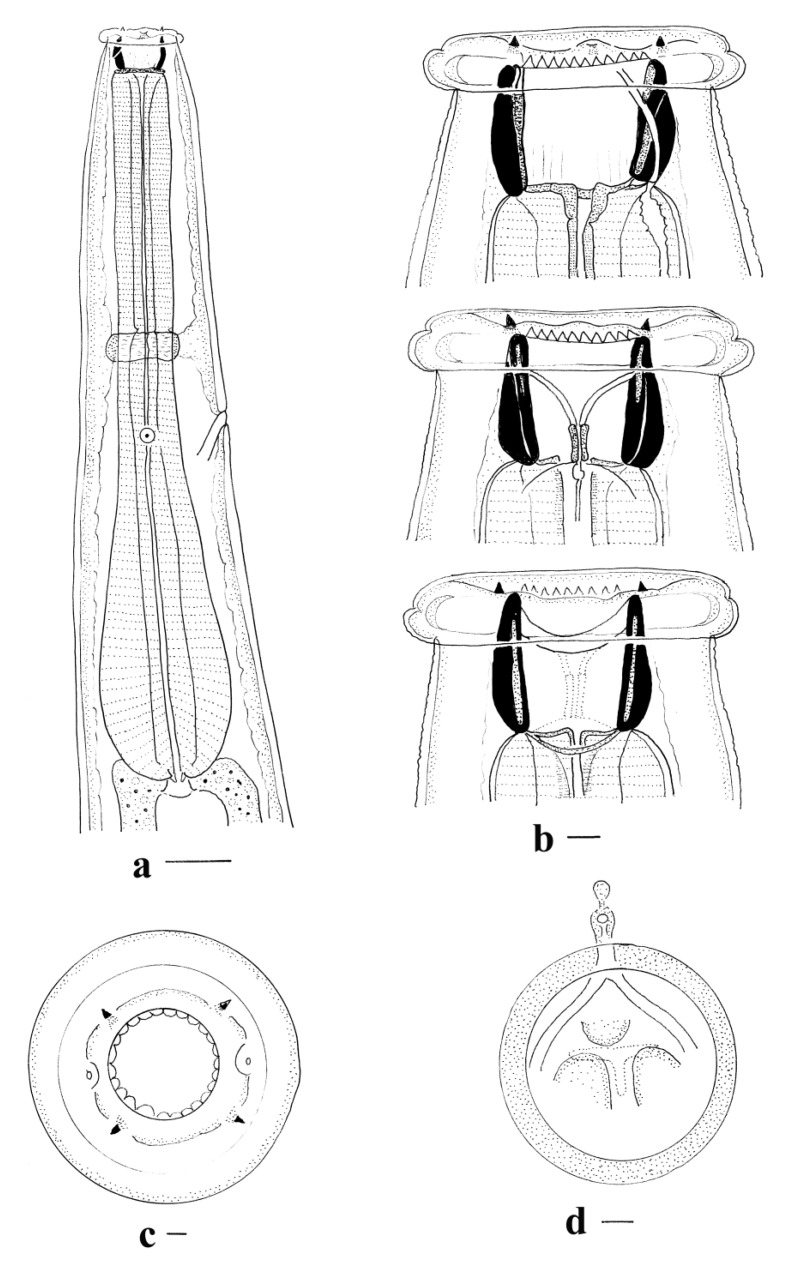
*Torquenema toraliforme* n. comb. (**a**) Anterior region, right lateral view; (**b**) Buccal capsule, dorsal and lateral views; (**c**) Anterior extremity, apical view; (**d**) Buccal capsule, optical transverse section. Scale-bars: (**a**), 0.1 mm; (**b**–**d**), 0.01 mm.

**Figure 2:**
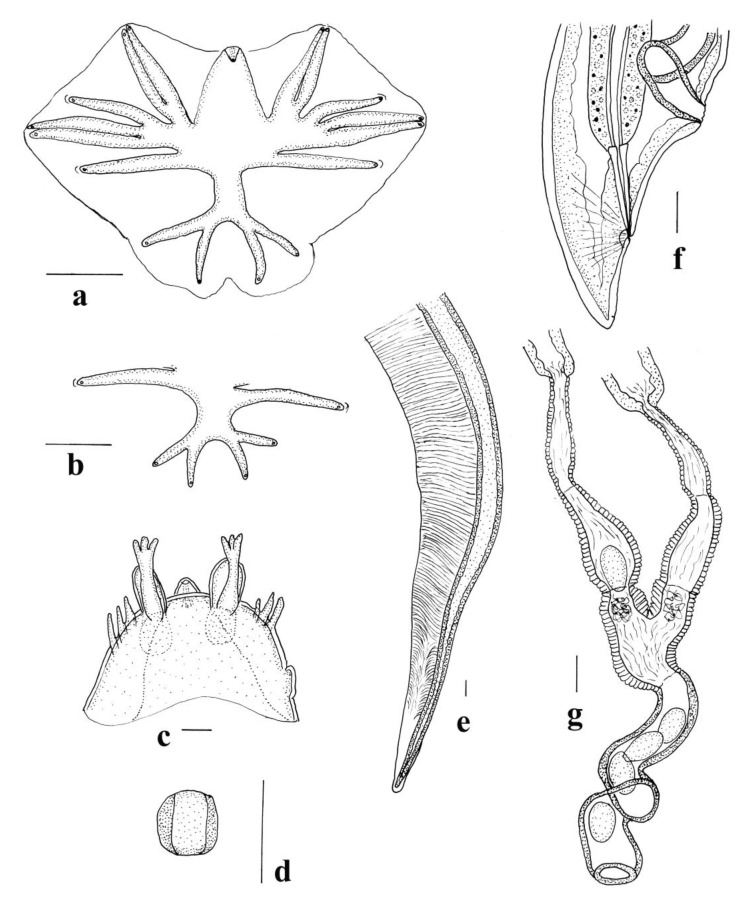
*Torquenema toraliforme* n. comb. (**a**) Bursa, apical view; (**b**) Dorsal ray of bursa, dorsal view; (**c**) Genital cone, dorsal view; (**d**) Gubernaculum; (**e**) Spicule tip, lateral view; (**f**) Female tail, lateral view; (**g**) Vagina and ovejector, lateral view. Scale-bars: (**a**,**b**,**f**–**g**), 0.1 mm; (**c**–**e**), 0.01 mm.

**Figure 3:**
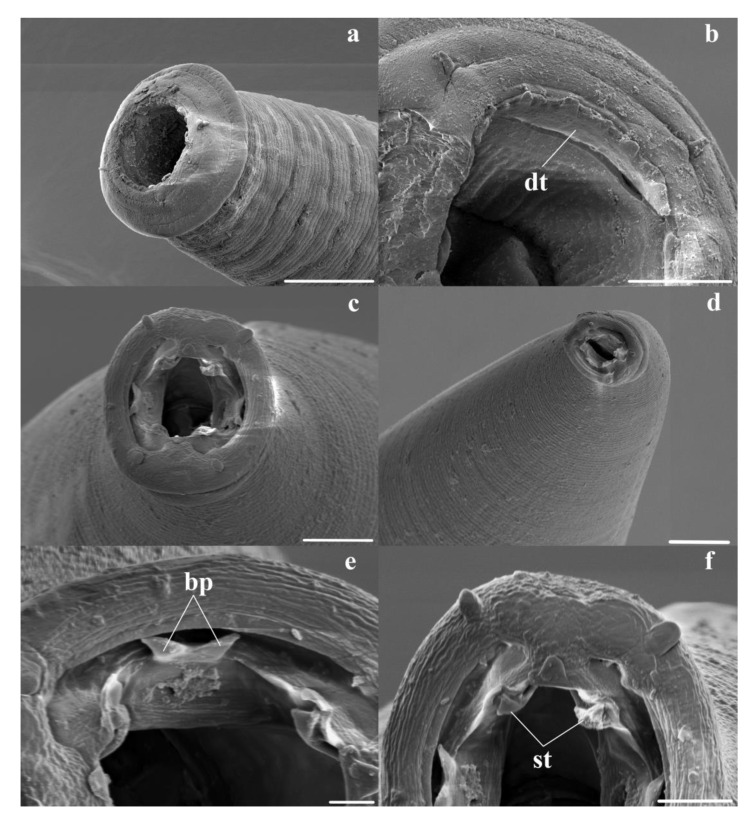
Scanning electron photomicrographs of the cephalic extremities and mouth openings. (**a**) Anterior extremity of *Torquenema toraliforme* n. comb. showing collar; (**b**) Buccal capsule, apical view of *Torquenema toraliforme* n. comb.(dt = denticles); (**c**) Buccal capsule, apical view of *Wallabicola dissimilis* n. comb.; (**d**) Anterior extremity of *Wallabicola dissimilis* n. comb., showing ventral deviation of head; (**e**,**f**) Buccal capsule, apical view of *Wallabicola dissimilis* n. comb. showing bifid projection (bp) (**e**) and sub-median teeth (st) (**f**). Scale-bars: (**a**), 50 µm; (**b**,**d**), 20 µm; (**c**), 10 µm; (**e**), 2 µm; (**f**), 5 µm.

*General* (Figure 1a–d, Figure 2a–g and Figure 3a,b). Robust nematodes, red or pink in colour when fresh. Body covered with numerous transverse annulations, c. 0.005 apart, with gaps between annulations along lateral surfaces of body. Cephalic collar enlarged, disc-shaped, extending peripherally beyond body; anterior surface divided by fine sutures visible with SEM, one surrounding cephalic papillae and amphids, outer suture close to peripheral margin (Figure 3a,b); outer region of collar with internal cavity, semi-lunar in shape in transverse section; inner region of collar pierced by four sub-conical, sub-medial papillae and two lateral amphids; mouth opening circular; buccal capsule sub-cylindrical, heavily sclerotised, longer than wide, with numerous inconspicuous internal longitudinal ridges, most readily visible in lateral views towards base of buccal capsule; anterior extremity bears c. 25 triangular projections forming leaf crown; teeth absent in buccal capsule; wall of buccal capsule complex with internal structure sub-divided; duct of dorsal oesophageal gland pierces wall of buccal capsule at mid-length, divides internally to form Y-shaped dorsal gutter, with branches passing internally near anterior margin of buccal capsule, joining ventrally. Oesophagus clavate; ventral sectors of oesophagus project prominently into base of buccal capsule; dorsal sector projects slightly into base of buccal capsule; nerve ring in anterior third of oesophagus; deirid papillate, posterior to nerve ring; excretory pore at about same level as deirid.

*Male (measurements of 10 specimens)*. Length 13–20 (16); maximum width 0.43–0.61 (0.53); buccal capsule 0.070–0.080 (0.078) deep, 0.060–0.070 (0.061) wide; width of cephalic collar 0.13–0.15 (0.14); oesophagus 0.88–1.26 (1.17) long; nerve ring, excretory pore and deirid 0.46–0.56 (0.52), 0.59–0.75 (0.67) and 0.63–0.78 (0.72) from anterior end respectively. Bursa short, stout; lateral and ventral lobes fused, ventral lobes joined ventrally; dorsal lobe shorter than ventro-lateral lobes, with slight median indentation. Ventro-ventral and ventro-lateral rays apposed, reaching margin of bursa; externo-lateral ray divergent from lateral trunk, not reaching margin of bursa, terminating in slight elevation on surface of bursa; medio-lateral and postero-lateral rays apposed, reaching margin of bursa; externo-lateral ray between lateral and dorsal trunks, not reaching margin of bursa, terminating in slight elevation on surface of bursa; dorsal ray slender at origin, dividing at mid-length; external branchlets directed postero-laterally, not reaching margin of bursa; internal branchlets arcuate, directed posteriorly, not reaching margin of bursa; spicules 2.95–3.45 (3.25) long, alate; alae with numerous transverse striations; anterior extremity of spicules irregularly knobbed; distal extremity curved, terminating in blunt tip; ala diminishes gradually towards distal tip of spicule, losing transverse striations; ventral lip of genital cone conical, bearing papilla 0; dorsal lip with paired appendages (papillae 7) closely applied to cloaca; dorsal to each of them, distally trifid projection; 3–4 elongate, simple projections lateral to each side of central projections; gubernaculum prominent, ovoid and laterally elongated in dorso-ventral view 0.13–0.15 (0.14) long.

*Female (measurements of 10 specimens).* Length 23–32 (27); maximum width 0.73–0.88 (0.79); buccal capsule 0.080–0.100 (0.090) deep, 0.006–0.007 (0.064) wide; width of cephalic collar 0.15–0.18 (0.16); oesophagus 1.28–1.60 (1.43) long; nerve ring, excretory pore and deirid 0.51–0.63 (0.58), 0.64–0.96 (0.78) and 0.64–0.99 (0.74) from anterior end respectively. Tail short conical, 0.24–0.30 (0.26); vulva 0.52–0.73 (0.60) from posterior end; vagina convoluted 0.50–0.61 (0.58) long; ovejector Y-shaped, c. 0.35 long; vestibule elongate, c. 0.36 long; infundibulum elongate with decreased muscular investment, c. 0.30 long; eggs thin-shelled, ellipsoidal 0.12–0.15 (0.14) long, 0.06–0.08 (0.07) wide.

#### 3.1.4. Remarks

*Torquenema* n. g. is distinguishable from all other phascolostrongyline genera by the presence of a large disc-shaped cephalic collar which protrudes beyond its body margin. *Torquenema* n. g. differs from *Paramacropostrongylus* by the Y-shaped ovejector and its occurrence within the caecum of the host. 

The current re-description of *T. toraliforme* n. comb. largely supports that of Beveridge and Mawson [2] and Beveridge [1], while providing additional details, particularly those shown by SEM. Beveridge and Mawson [2] reported the mouth opening of *P. toraliformis* as being surrounded by about 20 ‘denticles’. Their apical view of the mouth opening (Figure 59 in Beveridge and Mawson [2]) shows 23 ‘denticles’ which is closer to the 25 estimated in the current description. However, the current SEM observations of *Torquenema* n. g. (Figure 3a,b) suggest that these ‘denticles’ are not separate entities but form a continuous structure around the anterior margin of the buccal capsule, resembling a leaf crown. Consequently, they have here been described as such. The extent to which this situation differs from *Paramacropostrongylus* will require additional SEM studies of this genus. 

One of the principal morphological features used to separate the Phascolostrongylinae from the Cloacininae has been the origin of the externo-dorsal ray, arising from the dorsal trunk in the Phascolostrongylinae and from the lateral trunk in the Cloacininae. However, in *Torquenema* n. g., the externo-dorsal ray arises between the lateral and dorsal trunks, a feature partially recognised by Beveridge and Mawson [2] in their definition of *Paramacropostrongylus* in which they state ‘externo-dorsal ray arising from dorsal ray or separately’. Although uncommon within the Cloacininae, Beveridge [8] described two species of *Cloacina* in which the externo-dorsal ray arises from the dorsal ray. The current description of the genital cone differs slightly from that illustrated by Beveridge and Mawson [2] in that their Figure 62 lacks the actual papillae 7 on the dorsal lip, which are overshadowed by the paired appendages dorsal to them. 

**Figure 4:**
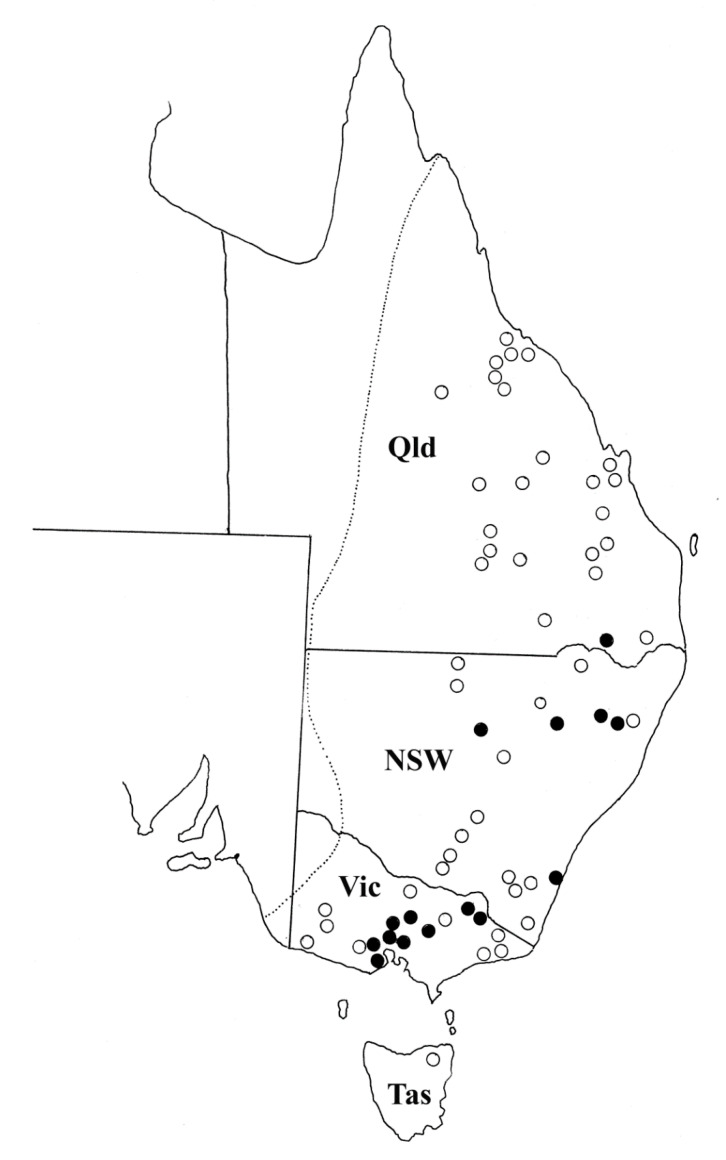
The geographic distribution of *Torquenema toraliforme* n. comb. The dotted line represents the distribution of the host, *Macropus giganteus* based on Van Dyck and Strahan [9]. Open circles represent locations in which hosts have been examined but *T. toraliforme* was absent. Solid circles represent locations in which *T. toraliforme* was found. Abbreviations; Qld = Queensland, NSW = New South Wales, Vic = Victoria, Tas = Tasmania.

The geographic distribution of *T. toraliforme* n. comb. corresponds to some degree with the distribution of its host, *M. giganteus* (Figure 4). Collection records show that *T. toraliforme* n. comb. is most commonly encountered in Victoria and some parts of New South Wales. However, *T. toraliforme* n. comb. appears to be absent from Queensland except for areas around Inglewood, close to the New South Wales border (Figure 4). 

### 3.2. Description of a New Genus, Wallabicola n. g.

Order Strongylida (Molin, 1861)

Family Chabertiidae (Popova, 1952)

Subfamily Phascolostrongylinae Lichtenfels, 1980

*Wallabicola* n. g.

*Type species*: *Wallabicola dissimilis* (Johnston and Mawson, 1939) n. comb.

#### 3.2.1. Generic Definition

Robust nematodes. Anterior extremity deviated slightly ventrally; cephalic collar prominent, pierced by four sub-conical, sub-median papillae and two lateral amphids; mouth opening elongated laterally; four triangular sub-median teeth arise from anterior wall of buccal capsule; buccal capsule sub-cylindrical, heavily sclerotised; duct of dorsal oesophageal gland runs within wall of buccal capsule but at mid-length, divides internally forming Y-shaped dorsal gutter. Oesophagus clavate; nerve ring in anterior third of oesophagus; deirid papillate, at level of nerve ring; excretory at pore same level as deirid and nerve ring. Spicules elongate, alate. Vulva immediately anterior to anus; ovejector J-shaped. Parasitic in macropodid marsupials.

#### 3.2.2. Redescription of *Wallabicola dissimilis n. comb.*

*Wallabicola dissimilis* (Johnston and Mawson, 1939) n. comb.

*Synonyms: Cyclostrongylus dissimilis* Johnston and Mawson, 1939;

*Macropostrongyloides dissimilis* (Johnston & Mawson, 1939) Mawson, 1977.

*Types*: holotype, SAM 42827. 

*Type locality*: Milson Island, New South Wales, Australia (33°31′ S 151°11′ E).

*Type host*: *Wallabia bicolor* (Desmarest,) (Marsupialia: Macropodidae).

*Additional material examined*: Queensland: 1♀, Toowoomba (SAM 6852); New South Wales: 1♀, Nowra (SAM 10959); 1♂, 1♀, Nadgee State Forest (SAM 47679); Victoria: 1♂, Mitta Mitta (SAM 12098); 5♂♂, 4♀♀, Kamarooka (SAM 6772, 30521); 1♂, 1♀, Buangor (SAM 33982, 34688); 1♂, 4♀♀, Hall’s Gap (SAM 45490); 3♂♂, 10♀♀, Mount Zero (SAM 44578); 11 ♂♂, 24♀♀, Brimpaen (SAM 44568); 1♂, 2♀♀, Bend of Islands (SAM 6773, 9259); 5♀♀, Healesville (SAM 32042); 1♀, Main Ridge (SAM 46300); 1♂, 2♀♀, Portland (SAM 46289); 2♂♂, 2♀♀, Traralgon South (SAM 19458); 9♂♂, 26♀♀, Bonang (SAM 6774, 7261, 10569, 10596); 1♀, Bemm River (SAM 6783).

*Site in host:* Stomach.

*Representative DNA sequences*: Ribosomal DNA first and second internal transcribed spacers sequences (Genbank Accession MK842126) from *W. bicolor*, Kamarooka, Victoria, Australia [5].

*Etymology*: The new generic name *Wallabicola* n. g. (masculine) is derived from ‘incola’ in Latin meaning inhabitant and *Wallabia* referring to the swamp wallaby, *Wallabia bicolor*, the host in which the new genus occurs.

#### 3.2.3. Redescription 

**Figure 5:**
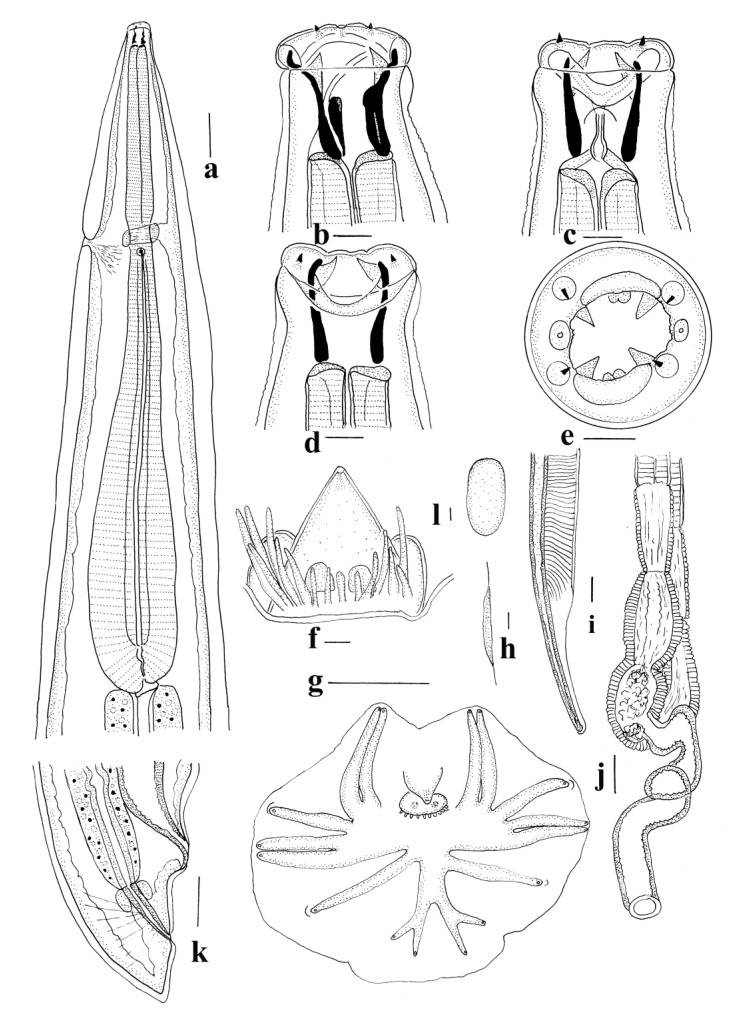
*Wallabicola dissimilis* n. comb. (**a**) Anterior region, lateral view; (**b**) Buccal capsule, lateral view; (**c**) Buccal capsule, dorsal view; (**d**) Buccal capsule, ventral view; (**e**) Buccal capsule, apical view; (**f**) Genital cone, dorsal view; (**g**) Bursa, apical view; (**h**) Gubernaculum, lateral view; (**i**) Spicule tip, lateral view; (**j**) Vagina and ovejector, lateral view; (**k**) Female tail, lateral view; (**l**) Gubernaculum, dorsal view. Scale-bars: (**a**,**g**,**j**), 0.1 mm; (**b**–**f**,**h**,**i**,**l**), 0.01 mm.

*General* (Figure 3c–f and Figure 5a–l). Robust nematodes, red in colour when fresh. Body covered with numerous, fine transverse annulations, c. 0.001 apart. Anterior extremity deviated slightly ventrally; cephalic collar prominent, pierced by four sub-conical, sub-medial papillae and two lateral amphids; mouth opening elongated laterally; cephalic collar projects into mouth opening adjacent to amphids, with apical inflection, membrane surrounding teeth joined; four triangular sub-median teeth arise from anterior wall of buccal capsule, project into lumen; membrane between teeth and wall of buccal capsule continuous with internal projections from amphidial region of cephalic collar and bifid dorsal and ventral projections on anterior margin of buccal capsule; buccal capsule sub-cylindrical, heavily sclerotised, longer than wide; duct of dorsal oesophageal gland runs within wall of buccal capsule but at mid-length, divides internally to form Y-shaped dorsal gutter, with branches passing internally near anterior margin of buccal capsule, joining ventrally. Oesophagus clavate; nerve ring in anterior third of oesophagus at slight oesophageal constriction; deirid papillate, at level of nerve ring; excretory pore at about same level as deirid and nerve ring.

*Male (measurements of 10 specimens).* Length 11–16 (13.6); maximum width 0.41–0.48 (0.45); buccal capsule 0.025–0.038 (0.029) deep, 0.018–0.023 (0.020) wide; oesophagus 0.89–1.10 (1.01) long; nerve ring, excretory pore and deirid 0.30–0.40 (0.35), 0.32–0.40 (0.36) and 0.34–0.41 (0.38) from anterior end respectively. Bursa short, stout; lateral and ventral lobes fused, ventral lobes joined ventrally; dorsal lobe shorter than ventro-lateral lobes, with slight median indentation. Ventro-ventral and ventro-lateral rays apposed, reaching margin of bursa; externo-lateral ray divergent from lateral trunk, not reaching margin of bursa, terminating in slight elevation on surface of bursa; medio-lateral and postero-lateral rays apposed, reaching margin of bursa; externo-lateral ray originating at base of dorsal trunk, not reaching margin of bursa, terminating in slight elevation on surface of bursa; dorsal ray slender at origin, dividing at mid-length; external branchlets directed postero-laterally, not reaching margin of bursa; internal branchlets directed posteriorly, not reaching margin of bursa; spicules 2.40–2.78 (2.53) long, alate; ala with numerous transverse striations; anterior extremity of spicules irregularly knobbed; distal extremity blunt; ala diminishes gradually towards distal tip of spicule, losing transverse striations; ventral lip of genital cone conical, bearing papilla 0; dorsal lip with paired appendages (papillae 7) closely applied to cloaca; dorsal to them array of elongate papillae; gubernaculum poorly sclerotised, ovoid and laterally elongated in dorso-ventral view 0.030–0.0.035 (0.032) long, slender in lateral views, not clearly visible in all specimens.

*Female* (measurements of 10 specimens). Length 16–24 (18.8); maximum width 0.49–0.56 (0.53); buccal capsule 0.020–0.030 (0.025) deep, 0.018–0.025 (0.020) wide; oesophagus 0.96–1.13 (1.05) long; nerve ring, excretory pore and deirid 0.34–0.40 (0.36), 0.34–0.43 (0.38) and 0.37–0.44 (0.41) from anterior end respectively. Tail short conical, 0.12–0.15 (0.13); vulva 0.29–0.39 (0.36) from posterior end; vagina slightly convoluted 0.40–0.52 (0.47) long; ovejector J-shaped; eggs thin-shelled, ellipsoidal 0.14–0.16 (0.15) long, 0.07–0.09 (0.08) wide.

#### 3.2.4. Remarks

*Wallabicola* n. g. most closely resembles *Macropostrongyloides*, differing in the J-shaped ovejector and its occurrence within the stomach of its host. The new genus is differentiated from other phascolostrongyline genera (except for *Hypodontus* and *Corollostrongylus*) by its ventrally deviated head. 

The current re-description of this species confirms most of the observations of Beveridge and Mawson [2] and Beveridge [1] while adding additional details of the spicule tip and cephalic structures. The description of the cephalic structures presented here differs significantly from that of Beveridge and Mawson [2], particularly in the position of the amphids. The cephalic extremity of *W. dissimilis* is extremely small and difficult to study under the light microscope. However, the scanning electron photomicrographs presented here provide novel information (Figure 3c–f). As with species of *Macropostrongyloides*, the buccal capsule contains four sub-median teeth with a flap-like structure resembling a leaf crown element arising between the tooth and the margin of the buccal capsule. Sukee et al. [10] speculated that these, along with the ‘denticles’ might constitute a leaf crown if they were continuous. The comparable structure in *W. dissimilis* is continuous although denticles are lacking. It differs however in that in *W. dissimilis* the structure includes bifid projections on the dorsal and ventral aspect of the margin of the buccal capsule, which are absent in species of *Macropostrongyloides*, and the lateral tridents seen in *Macropostrongyloides* are replaced by internal projections of the cephalic collar. The peri-oral structures of *W. dissimilis* are therefore quite distinct from those found in *Macropostrongyloides baylisi, M. mawsonae, M. spearei and M. woodi*, but the observations presented here support the suggestion of Sukee et al. [10] that an unusual form of leaf crown is present at the anterior margin of the buccal capsule in these species.

The female genital system of *W. dissimilis* was re-described by Beveridge (Figure 3A, [1]), but was not shown it in its entirety. The entire system is shown in Figure 5j. The vagina is slightly convoluted with the degree of convolution differing between specimens. 

**Figure 6:**
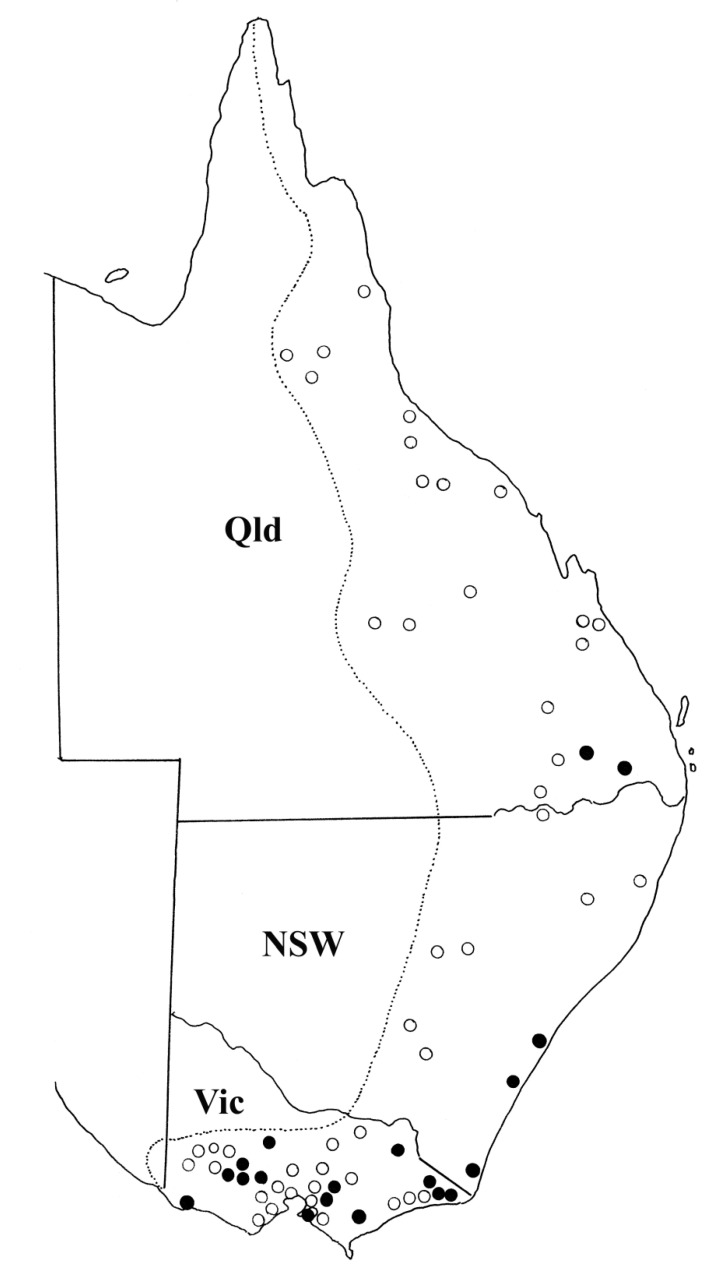
The geographic distribution of *Wallabicola dissimilis* n. comb. The dotted line represents the distribution of the host, *Wallabia bicolor* based on Van Dyck and Strahan [9]. Open circles represent locations in which hosts have been examined but *W. dissimilis* was absent. Solid circles represent locations in which *W. dissimilis* were found. Abbreviations; Qld = Queensland, NSW = New South Wales, Vic = Victoria.

The new genus is predominantly distributed in Victoria and New South Wales (Figure 6). Although the distribution of *W. bicolor* is continuous along the eastern coast of Australia from Victoria to far north Queensland, *W. dissimilis* is has rarely been encountered in Queensland [2,11].

### 3.3. Description of a New Species, Macropostrongyloides phascolomys n. sp.

*Macropostrongyloides phascolomys* n. sp. 

*Synonyms: Macropostrongylus lasiorhini* Mawson, 1955; *Macropostrongyloides lasiorhini* (Mawson, 1955) Yamaguti, 1961 (in part). 

*Type specimens*: holotype ♂, (SAM 49056); allotype ♀, (SAM 59057); paratypes 3♂♂, 1♀, (SAM 30361).

*Type host*: *Vombatus ursinus* (Shaw) (Marsupialia: Vombatidae).

*Type locality*: Bullengarook, Victoria, Australia (37°31′ S 144°29′ E).

*Additional material examined*: from *Vombatus ursinus*: New South Wales: 5♂♂, 20♀♀, Coolangubra State Forest (N 1511); 12♂♂, 22♀♀, composite collection from Bondo State Forest, Nimmo and Tharwa (N 1116); Victoria: 2♂♂, 4♀♀, Mount Pinnebar (SAM 13924).

*Site in host:* Colon.

*Representative DNA sequence*: Ribosomal DNA first and second internal transcribed spacers sequences (Genbank Accession MK842123) from *V. ursinus*, Bullengarook, Victoria, Australia [5].

*Etymology*: The new species is named after the former generic name of the host, *Phascolomys.*

#### 3.3.1. Description 

**Figure 7:**
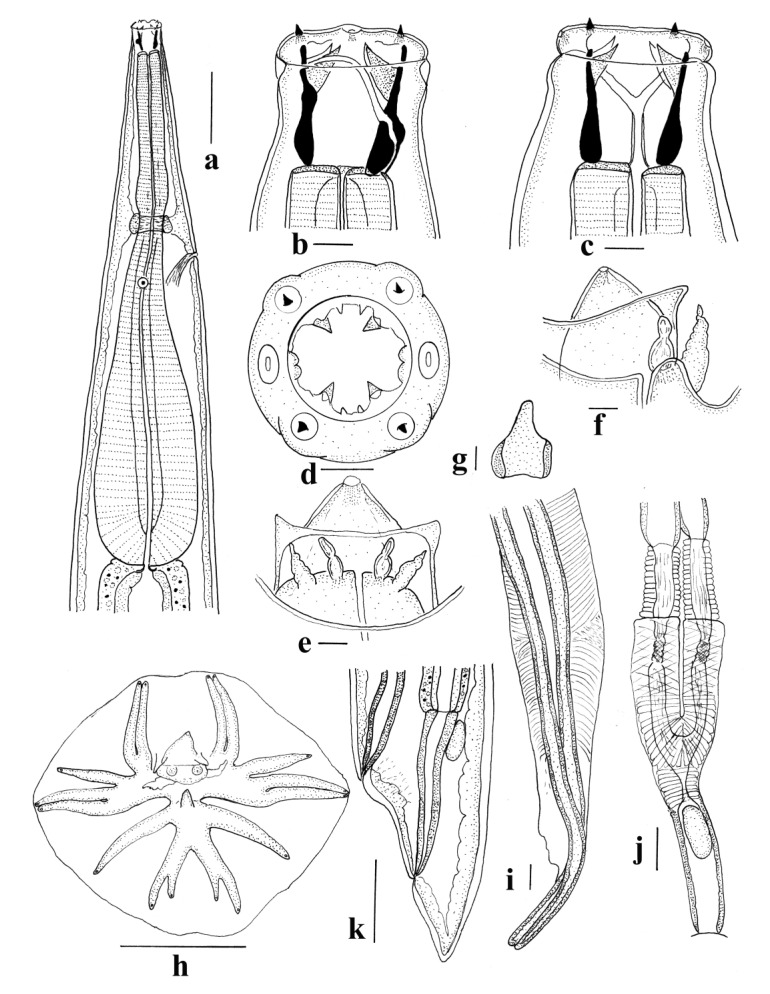
*Macropostrongyloides phascolomys* n. sp. (**a**) Anterior region, lateral view; (**b**) Buccal capsule, lateral view; (**c**) Buccal capsule, dorsal view; (**d**) Mouth, apical view; (**e**) Genital cone, dorsal view; (**f**) Genital cone, lateral view; (**g**) Gubernaculum, dorsal view; (**h**) Bursa, apical view; (**i**) Spicule tip, lateral view; (**j**) Vagina and ovejector, lateral view; (**k**) Female tail, lateral view. Scale-bars: (**a**,**e**–**h**,**j**–**k**), 0.1 mm; (**b**–**d**,**i**), 0.01 mm.

*General* (Figure 7a–k). Robust nematodes, pink in colour when fresh. Body without transverse annulations. Anterior extremity with prominent cephalic collar, pierced by four sub-conical, sub-medial papillae and two lateral amphids; mouth opening circular; four triangular sub-median teeth arise from anterior wall of buccal capsule, project into lumen; petaloid membrane present between teeth and wall of buccal capsule; bifid or trifid membranous structure present at anterior margin of buccal capsule, internal to each amphid; paired denticles present on anterior margin of buccal capsule dorsally and ventrally; buccal capsule sub-cylindrical, heavily sclerotised, longer than wide; duct of dorsal oesophageal gland runs within wall of buccal capsule but at mid-length, divides internally to form Y-shaped dorsal gutter, with branches passing internally near anterior margin of buccal capsule, joining ventrally. Oesophagus clavate; nerve ring in anterior third of oesophagus at slight oesophageal constriction; deirid papillate, at level of nerve ring; excretory pore at about same level as deirid and nerve ring.

*Male (measurements of 10 specimens).* Length 10–14 (11.7); maximum width 0.32–0.43 (0.38); buccal capsule 0.028–0.038 (0.033) deep, 0.025–0.030 (0.028) wide; oesophagus 0.71–0.82 (0.77) long; nerve ring, excretory pore and deirid 0.29–0.36 (0.32), 0.31–0.38 (0.35) and 0.35–0.45 (0.39) from anterior end respectively. Bursa short, stout; lateral and ventral lobes fused, ventral lobes joined ventrally; dorsal lobe shorter than ventro-lateral lobes, without median indentation. Ventro-ventral and ventro-lateral rays apposed, reaching margin of bursa; externo-lateral ray divergent from lateral trunk, not reaching margin of bursa; medio-lateral and postero-lateral rays apposed, reaching margin of bursa; externo-lateral ray originating at base of dorsal trunk, not reaching margin of bursa; dorsal ray slender at origin, dividing almost at origin; external branchlets directed posteriorly, not reaching margin of bursa; internal branchlets shorter, directed posteriorly, not reaching margin of bursa; spicules 1.00–1.18 (1.11) long, alate; ala with numerous transverse striations; anterior extremity of spicules irregularly knobbed; distal extremity blunt; ala diminishes abruptly towards distal tip of spicule, losing transverse striations; ventral lip of genital cone conical, bearing papilla 0; ridge ventral to lip bears two lateral projections; dorsal lip with paired appendages (papillae 7) closely applied to cloaca; lateral to them single elongate papilla projects from base of cone; prominent conical projection present in mid-line of internal surface of dorsal lobe; gubernaculum prominent, elongate with posterior section broader in dorso-ventral view 0.025–0.045 (0.035) long, slender in lateral views.

*Female (measurements of 10 specimens).* Length 14–18 (16.0); maximum width 0.45–0.69 (0.61); buccal capsule 0.030–0.040 (0.035) deep, 0.025–0.030 (0.028) wide; oesophagus 0.78–1.13 (1.02) long; nerve ring, excretory pore and deirid 0.31–0.40 (0.35), 0.29–0.43 (0.35) and 0.30–0.44 (0.38) from anterior end respectively. Tail short conical, 0.09–0.13 (0.11); vulva 0.22–0.31 (0.27) from posterior end; vagina straight, 0.38–0.48 (0.42) long; ovejector Y-shaped; eggs thin-shelled, ellipsoidal 0.12–0.16 (0.14) long, 0.04–0.07 (0.06) wide.

#### 3.3.2. Remarks 

**Figure 8:**
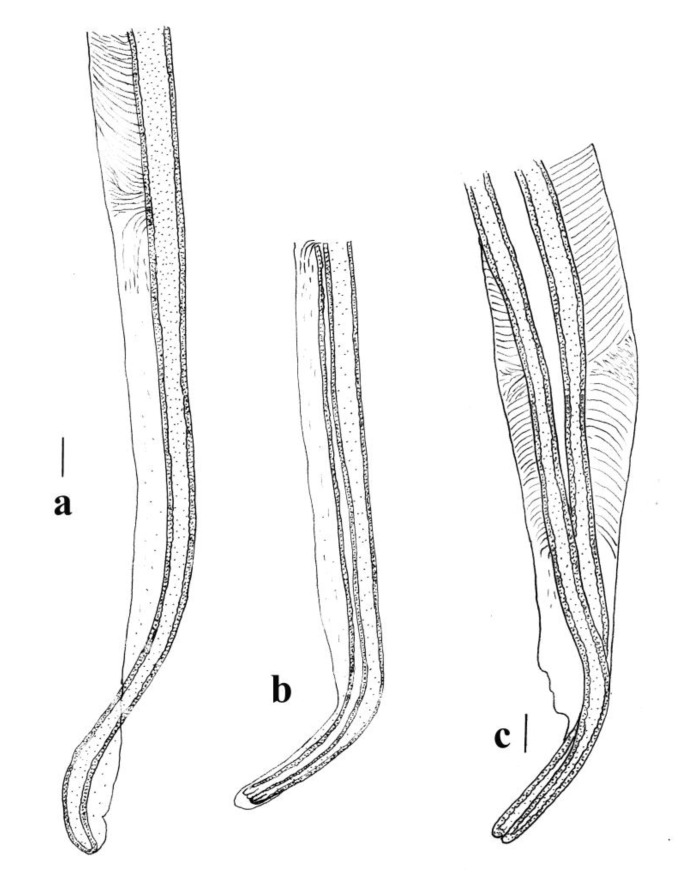
Comparative views of the spicule tips of *Macropostrongyloides lasiorhini* and *Macropostrongyloides phascolomys* n.sp. (**a**) Spicule tip of *Macropostrongyloides lasiorhini*, lateral view; (**b**) Spicule tip of *Macropostrongyloides lasiorhini*, oblique view; (**c**) Spicule tip of *Macropostrongyloides phascolomys* n. sp., lateral view. Scale-bars: 0.01 mm.

The new species is very similar to *M. lasiorhini* with which it has been confused in the past. The females of the two species are indistinguishable and the only character separating the males is the morphology of the genital cone which has a ridge ventral to the ventral lobe of the cone and a pair of lateral projections on the two sides of the base of the dorsal part of the cone. In *M. lasiorhini*, the dorsal lobe of the cone is surrounded by numerous projections, some of which are bifid terminally. The spicule tip of *M. lasiorhini* has not been described in detail previously but it is distinguishable from the spicule tip of *M. phascolomys* n. sp. The comparisons between the spicule tips of *M. lasiorhini* and *M. phascolomys* n. sp. are shown in Figure 8a–c. There is a slight bend in the spicule tip of *M. phascolomys* n. sp. compared to the almost 45 degree bend in the spicule tip of *M. lasiorhini* (Figure 8b,c). The spicule ala in *M. phascolomys* terminates abruptly prior to reaching the end of spicule tip whereas in *M. lasiorhini,* the ala continues toward the end (Figure 8a) and wraps around the spicule tip in oblique views (Figure 8b). The striation patterns also differ between the two species, in *M. lasiorhini* the striation patterns change orientation further from the spicule tip and diminishes completely (Figure 8a,b) whereas in *M. phascolomys* n. sp., after the change in orientation the striations continue until almost reaching the end of the ala (Figure 8c).

The wombat host species of *M. phascolomys* n. sp. and *M. lasiorhini* also differ, with the former species occurring in *V. ursinus* and the latter in *L. latifrons*.

## 4. Discussion 

Examination of specimens previously identified as *P. toraliformis* and *M. dissimilis* revealed differential morphological features which warrant the transfer of these species into two new genera, *Torquenema* n. g. and *Wallabicola* n. g., respectively. The study also found that *M. lasiorhini* from *V. ursinus* varied from specimens in the type-host, *L. latifrons*; therefore, permitting the description of a new species *M. phascolomys* n. sp., which is restricted to *V. ursinus*. The new genera and species described here are supported by previous molecular data [3,5]. 

Whilst the current descriptions of the genera *Torquenema* and *Wallabincola* are largely consistent with previous work by Beveridge and Mawson [2], additional informative characters have been added to the current descriptions. The structure of the deirids is relatively uniform throughout the species of the Phascolostrongylinae and is short and papillate in shape. In the Cloacininae, the deirid is elongate and hair-like, and this difference may provide a means of separating the two sub-families. The structure was overlooked in previous taxonomic descriptions. The use of scanning electron microscopy has enabled detailed observations of the peri-oral structures, including the flap-like projections associated with the teeth of *W. dissimilis* and denticles of *T. toraliforme*. A previous study of *Macropostrongyloides* spp. observed similar structures and speculated that the peri-oral projections may be leaf-crowns given that they extend the full length of the buccal capsule [10]. The SE photomicrographs of the mouth opening of *T. toraliforme* (Figure 3b) and *W. dissimilis* (Figure 3c,e,f) show that these peri-oral structures in both species appeared to be continuous around the buccal capsule rather than projecting as individual elements, suggesting that the structures could potentially be considered leaf-crowns. The presence of leaf-crown elements is characteristic of some genera of the subfamily Cloacininae and has only been observed in the subfamily Phascolostrongylinae in the genera *Phascolostrongylus* and *Oesophagostomoides* from wombats [1]. Whether this is also the case in other genera and species within the Phascolostrongylinae is not known and additional SEM examination is required, particularly of *Paramacropostrongylus, Macropostrongyloides yamagutii* and *M. lasiorhini* which possess both teeth and denticles. 

The shapes of the ovejector were used to differentiate the genera *Torquenema* n. g. and *Wallabincola* n. g. from their former genera. The presence of a Y-shape muscular ovejector is considered a plesiomorphic character within the Strongyloidea [12,13]. The genus *Torquenema* n. g. is differentiated from the genus *Paramacropostrongylus* by the presence of a Y-shaped ovejector and the genus *Wallabincola* is distinguished from *Macropostrongyloides* based on its J-shaped ovejector. In this study, the shape of the ovejector has been used as a generic character. However, the shape of the ovejector is not always reliable as a generic character. In the genus *Cloacina* for instance, species possess either a Y-shaped or J-shaped ovejector [14].

The geographic distribution of *T. toraliforme* and *W*. *dissimilis* appears to be primarily in the southern region of the distributions of their hosts (Figure 4 and Figure 6). Similar patterns have been observed in *Macropostrongyloides mawsonae* from *M. giganteus* which is predominantly encountered in the southern region of Australia but rarely detected in the northern part of Australia despite the continuous distribution of the host [10]. Previous molecular studies [3,5] have indicated genetic differences between specimens of *W. dissimilis* from Queensland and those collected from Victoria. No morphological differences were detected between these two genotypes in the current study, but the existence of a cryptic species cannot be eliminated. *Torquenema* n. g. occurs primarily in *M. giganteus* in Victoria whereas *Paramacropostrongylus iugalis* occurs in the same host in northern New South Wales and central Queensland [15]. 

*Macropostrongyloides phascolomys* n. sp. occurs in *V. ursinus* while *M. lasiorhini,* occurs in *L. latifrons.* Fossil records show that the wombat genus *Lasiorhinus* once had a wider distribution and was present in Victoria, New South Wales in Queensland between the late-Miocene to mid-Pliocene period [16]. This suggests that in the past, *Lasiorhinus* and *Vombatus* may have been sympatric while the current geographic distribution of *M. lasiorhini* and *V. ursinus* is allopatric. Therefore, the two species of *Macropostrongyloides* may have undergone co-speciation with their respective hosts. 

## 5. Conclusions

The present study described two new genera and a new species from the subfamily Phascolostrongylinae supporting previous molecular evidence [3,5,17]. Future studies utilising the nuclear and mitochondrial DNA sequence data could provide better insights into the evolution of this highly diverse group of parasitic nematodes from herbivorous Australian marsupials.

## Data Availability

This article was registered under the Official Register of Zoological Nomenclature (ZooBank) as urn:lsid:zoobank.org:pub:F9E8C1F1-2C75-4B75-BF70-7888599B5C7B. The new genera *Torquenema* and *Wallabicola* were registered as urn:lsid:zoobank.org:act:0CF562C1-BF53-4C9B-99C4-03D35FEC99A4 and urn:lsid:zoobank.org:act:06678CBD-5F42-4424-93D8-8CF09C664F91, respectively. The new species *Macropostrongyloides phascolomys* was registered as urn:lsid:zoobank.org:act:2FB8A60F-8748-4D42-A7C8-BE1327FC6051.

## References

[1] Beveridge I. (1987). The systematic status of Australian Strongyloidea (Nematoda). Bull. Mus. Natl. Hist. Nat..

[2] Beveridge I., Mawson P.M. (1978). A taxonomic revision of the genera *Macropostrongyloides* Yamaguti and *Paramacropostrongylus* Johnston & Mawson (Nematoda: Trichonematidae) from Australian marsupials. Aust. J. Zool..

[3] Sukee T., Beveridge I., Sabir A.J., Jabbar A. (2021). Phylogenetic relationships within the nematode subfamily Phascolostrongylinae (Nematoda: Strongyloidea) from Australian macropodid and vombatid marsupials. Microorganisms.

[4] Mawson P.M. (1997). Revision of the genus *Macropostrongylus* and descriptions of three new genera: *Popovastrongylus, Dorcopsinema* and *Arundelia* (Nematoda: Trichonematidae). Trans. R. Soc. S. Aust..

[5] Sukee T., Beveridge I., Chilton N.B., Jabbar A. (2019). Genetic variation within the genus *Macropostrongyloides* (Nematoda: Strongyloidea) from Australian macropodid and vombatid marsupials. Parasitology.

[6] Chabaud A.G., Puylaert F., Bain O., Petter A.J., Durette-Desset M.C. (1970). Remarques sur l’ homologie entre les papilles cloacales des Rhabditides et les côtes dorsales des Strongylida. C. R. Hebd. Séances Acad. Sci..

[7] Jackson S., Groves C. (2015). Taxonomy of Australian mammals.

[8] Beveridge I. (2014). New species of parasitic nematodes of the genus *Cloacina* (Nematoda: Strongyloidea) from the banded hare wallaby, *Lagostrophus fasciatus* (Marsupialia: Macropodidae). Trans. R. Soc. S. Aust..

[9] Van Dyck S., Strahan R. (2008). The Mammals of Australia.

[10] Sukee T., Beveridge I., Jabbar A. (2020). New species of *Macropostrongyloides* Yamaguti, 1961 (Nematoda: Strongylida) and the redescription of *Ma. baylisi* (Wood, 1930) from Australian macropodid marsupials. Syst. Parasitol..

[11] Beveridge I. (2016). The gastro-intestinal helminth parasites of the swamp wallaby, *Wallabia bicolor* (Desmarest) (Marsupialia: Macropodidae), and their regional distribution. Trans. R. Soc. S. Aust..

[12] Lichtenfels J.R. (1979). A conventional approach to a new classification of the Strongyloidea, nematode parasites of mammals. Am. Zool..

[13] Lichtenfels J.R., Anderson R.C., Chabaud A.G., Willmott S. (1980). Keys to genera of the superfamily Strongyloidea No. 7. CIH Keys to the Nematode Parasites of Vertebrates.

[14] Beveridge I. (1998). Taxonomic revision of the genus *Cloacina* von Linstow (Nematoda: Strongyloidea) from macropodid marsupials. Invertebr. Syst..

[15] Chilton N.B., Beveridge I., Andrews R. (1993). Electrophoretic and morphological analysis of *Paramacropostrongylus typicus* (Nematoda: Strongyloidea), with the description of a new species, *Paramacropostrongylus iugalis*, from the eastern grey kangaroo *Macropus giganteus*. Syst. Parasitol..

[16] Murray P.F., Wells R.T., Pridmore P.A. (1998). Palaeontology and palaeobiology of wombats. Wombats.

[17] Sukee T., Koehler A.V., Hall R., Beveridge I., Gasser R.B., Jabbar A. (2020). Phylogenetic analysis of mitogenomic data sets resolves the relationship of seven *Macropostrongyloides* species from Australian macropodid and vombatid marsupials. Pathogens.

